# Photogrammetry-based stereoscopic optode registration method for functional near-infrared spectroscopy

**DOI:** 10.1117/1.JBO.25.9.095001

**Published:** 2020-09-02

**Authors:** Xiao-Su Hu, Neelima Wagley, Akemi Tsutsumi Rioboo, Alexandre F. DaSilva, Ioulia Kovelman

**Affiliations:** a University of Michigan, School of Dentistry, Department of Biologic and Materials Sciences and Prosthodontics, Headache & Orofacial Pain Effort (H.O.P.E.) Lab, Ann Arbor, Michigan, United States; b University of Michigan, Center for Human Growth and Development, Ann Arbor, Michigan, United States; c University of Michigan, Department of Psychology, Ann Arbor, Michigan, United States

**Keywords:** optode/probe registration, functional near-infrared spectroscopy, photogrammetry, magnetic resonance imaging

## Abstract

**Significance:** Functional near-infrared spectroscopy (fNIRS) is an emerging brain imaging technique due to its small size, low cost, minimum scanning sonic noise, and portability. Unfortunately, because this technique does not provide neuroanatomical information to accompany the functional data, its data interpretation remains a persistent challenge in fNIRS brain imaging applications. The two most popular approaches for fNIRS anatomical registration are magnetic resonance imaging (MRI) and three-dimensional (3-D) digitization. MRI scanning yields high-precision registration but reduces the cost-effectiveness and accessibility of fNIRS imaging. Alternatively, the low cost and portable 3-D digitizers are affected by magnetic properties of ambient metal objects, including participant clothing, testing equipment, medical implants, and so forth.

**Aim:** To overcome these obstacles and provide accessible and reliable neuroanatomical registration for fNIRS imaging, we developed and explored a photogrammetry optode registration (POR) method.

**Approach:** The POR method uses a consumer-grade camera to reconstruct a 3-D image of the fNIRS optode-set, including light emitters and detectors, on a participant’s head. This reconstruction process uses a linear-time incremental structure from motion (LTI-SfM) algorithm, based on 100 to 150 digital photos. The POR method then aligns the reconstructed image with an anatomical template of the brain.

**Results:** To validate this method, we tested 22 adult and 19 child participants using the POR method and MRI imaging. The results comparisons suggest on average 55% and 46% overlap across all data channel measurements registered by the two methods in adult and children, respectively. Importantly, this overlap reached 65% and 60% in only the frontal channels.

**Conclusions:** These results suggested that the mismatch in registration was partially due to higher variation in backward optode placement rather than the registration efficacy. Therefore, the photo-based registration method can offer an accessible and reliable approach to neuroanatomical registration of fNIRS as well as other surface-based neuroimaging and neuromodulation methods.

## 1 Introduction

Functional near-infrared spectroscopy (fNIRS) is a brain imaging technique that detects variations in oxyhemoglobin (HbO) and deoxyhemoglobin (HbR) concentration,^1^ comparable to the blood oxygen level-dependent signal.^2^ The portability and compatibility of ferromagnetic/electrical components of the fNIRS device provide researchers the flexibility to use localized brain imaging across varying experimental settings or populations, especially those in which high-resolution functional magnetic resonance imaging (fMRI) cannot be applied. These applications, for example, included the study of auditory plasticity,^3^ language development,^4^ cochlear implanted recipients,^5^ and pain in clinical environments.^6^^,^^7^ Yet, even though fNIRS can provide head surface-based spatial information for its probes, it has limitations in localizing anatomical brain regions that generate the hemodynamic response. Without accurate and consistent probe location estimates, variation in the collected functional data may not reflect true differences in underlying neural function as expected; instead, it could be a result of recordings from different cortical regions.

There are three localization methods commonly used in fNIRS neuroimaging: 10-20 reference system, magnetic resonance imaging (MRI) scan, and three-dimensional (3-D) digitizer.^8^^–^^11^ The extensively validated 10-20 reference system was developed for electroencephalograph (EEG) scalp electrode positioning.^12^ It is an essential first step in designing fNIRS headcaps as it provides researchers with a yardstick for building optode placements maximally overlaying neuroanatomical regions of interest.^9^^,^^11^ The 10-20 system also helps researchers properly position the fNIRS headcaps on participants’ heads. Yet, because the optode distances must remain fixed, fNIRS caps do not stretch in a way as EEG caps, even when they are adjusted for individual head sizes. Thus the main shortcoming of the EEG registration for fNIRS is the lack of more individualized localization. In summary, while the 10-20 system is a reliable first-step approach to fNIRS probe-set design and head placement, a complimentary approach is necessary to yield individual-level neuroanatomical precision.

One of the most effective methods is to co-register individual fNIRS data to his/her structural MRI using vitamin E capsules as fiducial markers.^8^^,^^10^^,^^13^ Given the high spatial resolution of MRI scanning, as well as the consideration of between-subject variability, this method yields some of the most reliable localization outcomes.^8^^,^^10^ However, MRI registration is costly, labor intensive, and limited to scanner availability, and it reduces the accessibility of the fNIRS method for studying young children, individuals with implants, and the like.^9^

Finally, a low-cost solution is the 3-D magnetic digitizer for fNIRS optode localization. This localization method pinpoints fNIRS optode locations onto a standard MRI scanned brain image with a magnetic stylus digitizer.^14^^,^^15^ Although this method does not acquire individualized neuroanatomical information, it accounts for the variation between participants.^9^^,^^10^^,^^16^ However, this method still requires the purchase of a specific device: a 3-D magnetic digitizer, which can be costly. Importantly, this device can be easily affected by the highly conductive material (e.g., steel) that is near or around the digitizer device, known as eddy distortion and permeability distortion.^17^ Such potential distortions in the digitization process limit the scenarios in which the digitizer-based registrations can be applied (e.g., in a room without metal furniture or equipment or only in patients without metal implants). Finally, digitized coordinates are usually analyzed after data collection, which can cost extra effort if digitization points are distorted and need to be recollected for each specific individual.

To overcome the obstacles that are associated with the current registration methods, we presented a photo-based photogrammetry optode registration (POR) method for fNIRS imaging in this paper. Photogrammetry is a cost-effective technique that uses a consumer-grade camera to provide accurate solutions in geography, architecture, and other domains requiring 3-D registration. This technique has been applied theoretically for EEG electrode registration in a previous study.^18^ In a previous study, Clausner et al. applied photogrammetry technology to register 68 EEG electrodes on a replica adult head to MRI space. They reconstructed a 3-D mesh head model based on a set of photos taken by a digital single-lens reflex camera and achieved a registration error of 0.8 mm, superior to an error of 6.1 mm yielded by 3-D digitization under the same setting. This EEG registration study suggested the possibility of high-precision localization using the photogrammetry technique. Following this study, we further evaluated the feasibility of the POR method for fNIRS optode registration by applying it to real adult and child participants. We included children in this study because the fNIRS methodology has been particularly popular with pediatric neuroimaging as an alternative to fMRI due to its child-friendly and portability features that allow the use of the system in school settings. It is, therefore, important that we examine the localization quality for both children and adults. In addition, to test whether the POR method can be applied in a cost-effective and convenient way, we employed a regular digital camera (iPad camera) in this study. Finally, and more importantly, to the best of our knowledge, this is the only paper to report a direct contrast between two surface-based registration methods along multiple dimensions as the performance of the POR method used alone was compared with and validated by MRI image-based registration.

## 2 Method

### 2.1 Participants

Twenty-two adults (mean age of 29.1 years, standard deviation 11.2 years, 5 females, average head conference 56.25 cm) and 19 children (mean age of 9.22 years, standard deviation 1.4 years, 16 females, average head conference 54.03 cm) participated in the study. Participation criteria included being healthy, right-handed, no metal implants, and no history of neurological diseases or brain damage. All participants signed the consent and age-appropriate assent or consent forms and received monetary compensation for their participation. The University of Michigan Institutional Review Board for ethical research approved the study.

### 2.2 fNIRS Probe Set Design

**Fig. 1:**
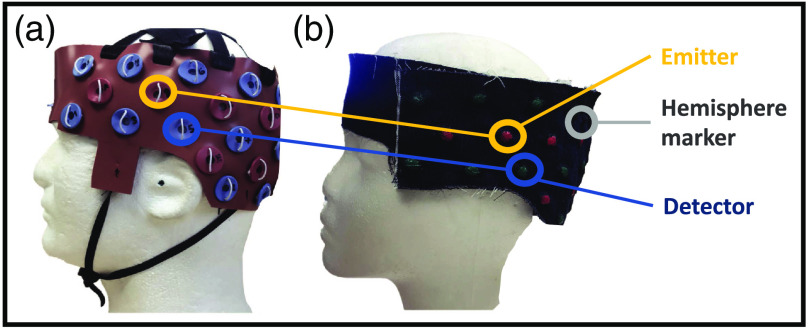
Cap design for the experiment. (a) The cap we used in daily fNIRS data collection. The cap contains 18 optodes (6 sources and 12 detectors), forming 23 channels per hemisphere. (b) A special-designed version of the data collection cap using denim, with vitamin E capsules imbedded for MRI scanning and color marker for photogrammetry.

The overall optode-set design was guided by our groups’ research interests in language processing, therefore covering lateral frontal and temporoparietal regions, bilaterally. For the purpose of testing the POR method, we designed a unilateral (left-hemisphere) cap to hold 6 light emitters and 12 light detectors on each hemisphere, based on the international 10-20 system,^19^ as shown in Fig. 1(b). These 18 optodes in total were arranged to yield 23 data channels or source–detector pairs at 2.8 cm away from each other. The original cap used for fNIRS imaging was made of silicone rubber with optode holders [Fig. 1(a)]. For this study, we designed an MRI-compatible version of this cap to contain vitamin-E capsules in place of the optode holders [Fig. 1(b)]. These capsules are used as markers because they contain fat that can be easily detected as signal intense by fMRI. The caps were built to accommodate different head sizes at 2-cm increments. Importantly, we marked the locations of the vitamin E capsules with bright green and pink colors to indicate emitter and detector positions, respectively, for the POR method.

### 2.3 Photogrammetry

We used an Apple iPad camera (8 megapixels, Model MW742LL/A, Apple Inc, CA) to take 100 to 150 photos per participant in continuous 360-deg rotation around the head. The total number of photos was estimated as follows: photos were taken roughly 0.8 m from the participants’ heads as study-team members took pictures and walked in a circle around the subjects with 0 to 40 deg of rotation. The photoreconstruction process, given an 8 megapixel iPad camera and ∼50% of photo-to-photo overlapping, required roughly 85 photos in total. Therefore, to ensure that our reconstructed 3-D image captures sufficient detail of the probe setup, we estimated that we would need to take 90 photos. We took additional photos, beyond the necessary 90, to account for the 10% to 40% of images that were blurry due to human error in either participant or experimenter movement. According to the linear-time incremental structure from motion (LIT-SfM) toolbox we used in this study, insufficient photos may lead to failed feature overlapping and dense reconstruction step, yielding a failed 3-D photo reconstruction. The photos were taken before and after the MRI session to account for a potential shift in the optode-set placement due to various head-related MRI preparation procedures, such as placements of motion-reducing and noise-cancelling gear around the participants’ heads. We selected the iPad camera because it is cost-effective and easy-to-use compared with digital single lens reflective cameras. The parameters like ISO value and aperture size were automatically set for every photo. The iPad cameras also allowed us to visually check the quality of every photo on the iPad screen in real time to make sure it qualified for the photoreconstruction process. We used pure color walls as screens to block the noises during the photosessions. This setup can be also achieved by a piece of cloth or curtain screen.

**Fig. 2:**
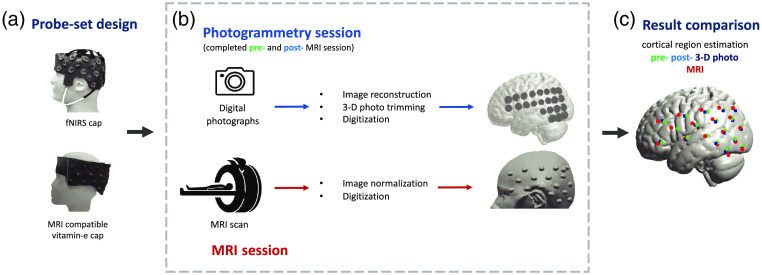
Experiment flowchart.

We used the LIT-SfM to reconstruct 3-D images of optode-set on the head surface based on the collected photos.^20^ The LIT-SfM algorithm requires only O(n) time complexity for the major reconstruction process, compared with $O(n^{4})$ required by the incremental SfM algorithm, where n refers to the number of photos in our study. Briefly, this method first calculates scale-invariant feature transform (SIFT) features for all collected photos. The SIFT feature is a set of image contents that are invariant to translation, rotation, scale, and other imaging parameters. For each photo, the algorithm then sorts these features into descending order by size and searches for matched feature pairs using a preemptive feature matching approach. Of the predefined features, only the largest of the two photos are examined and matched for speed optimization. Finally, the information in each single photo is integrated into the reconstructed 3-D image sequentially and a bundle adjustment procedure was implemented to decrease projection error. Practically, we ran the LIT-SfM algorithm using VisualSFM toolbox^21^ in this study. For each participant, we then used Meshlab software^22^ to generate a finite-element mesh head object (3-D photo) with the predesigned optode-set based on the 3-D image generated by the SfM algorithm. Finally, we loaded the 3-D photo into MATLAB (Mathworks, MA, USA) and pinpointed the positions of sources and detectors with five reference points (nasion, inion, Cz, and bilateral periarticular points). These emitter and detector positions were affinely transferred into the MNI space using a software package AtlasViewerGUI.^14^ We then projected these optode locations from the head surface onto the cortical surface of a MNI152 nonlinear brain template^23^ using the PullPtsToSurf function in the AtlasViewGUI toolbox. This process was repeated twice, for the pre- and post-MRI scanning images. A complete flowchart can be found in Fig. 2.

### 2.4 MRI Scan

Each participant was scanned with a 3.0-T GE MRI scanner (MR750, DV25.0 software version). For anatomical imaging, we used a 3-D volume spoiled gradient-echo pulse sequence ($0.469\;{\mathrm{mm}}^{2}$) in the axial plane and obtained 1.3-mm-thick slices. We used SPM12 to reorient the acquired images and then normalized them to the MNI 152 nonlinear template that was created by nonlinear registration of 152 T1-weighted images. We then manually localized the optode positions with the normalized images displayed in software MRIcron.^24^ Like the POR method, we then projected the probe locations from the head surface onto the cortical surface using the pullPtsToSurf function in the AtlasViewGUI toolbox. This optode registration process is similar to those used in prior works, such as Ref. 13, see also Fig. 2.

### 2.5 Data Analysis and Comparison Metrics

For data analyses, the orientation of the three axes was defined as follows: x axis pointed toward left (negative) and right (positive) hemispheres; y axis pointed toward anterior (positive) and posterior (negative) brain; and z axis pointed toward the dorsal (positive) and ventral (negative) brain.

We employed a total of five analytical steps and metrics for registration method comparison. First, we estimated the location for each optode for each person in MNI space. Second, we estimated a between-participant difference in optode placement for each method. To accomplish this, the composite standard deviation (Csd) was calculated as $\mathrm{Csd}=\sqrt{sd_{x}^{2}+sd_{y}^{2}+sd_{z}^{2}}$ for each optode, where $sd_{x}$, $sd_{y}$, and $sd_{z}$ represent deviations along the x, y, and z axes, respectively.^25^ Third, we calculated the optode-specific distance between the positions estimated through the two methods. Fourth, we calculated the overlap between the POR and MRI methods for each of the data channels. This was done by overlapping two spheres with a radius of 20 mm centered at each channel. fNIRS data channels are defined as the midpoint between the source–detector optode pairs. In the fifth and final method, we estimated the brain region that best corresponded to the individual data channels for each registration method separately. We compared the detectable regions of each fNIRS data channel estimated through the POR and MRI methods using Monte Carlo simulation based on a spherical model. Specifically, we defined a spherical space as the bounding box for a random sampling process; this bounding box can be described as: ${\left(x-x_{\mathrm{ch}}\right)}^{2}+{\left(y-y_{\mathrm{ch}}\right)}^{2}+{\left(z-z_{\mathrm{ch}}\right)}^{2}=r^{2}$, where r is the radius of the sphere. The center of the spherical space was placed at the mid-point between each source–detector pair (channels $x_{\mathrm{ch}}$, $y_{\mathrm{ch}}$, and $z_{\mathrm{ch}}$). We then randomly sampled 10,000 particles within the bounding box (r=10  mm). Each sampled point’s MNI coordinates were then matched to the corresponding brain region. We used the database in xjView toolbox (WFU_PickAtlas according to the xjView documentation^26^) for the matching process. Finally, to make a list we tabulated the obtained brain regions for each channel.

In this study, we used this adult MNI template for both adult and pediatric populations’ registration given that the average age of the children studied here is 9 years old. Previous MRI studies have confirmed such an application, specifically that a child’s brain is very similar to an adult brain at or above age 6 years old.^27^^,^^28^

## 3 Results

**Fig. 3:**
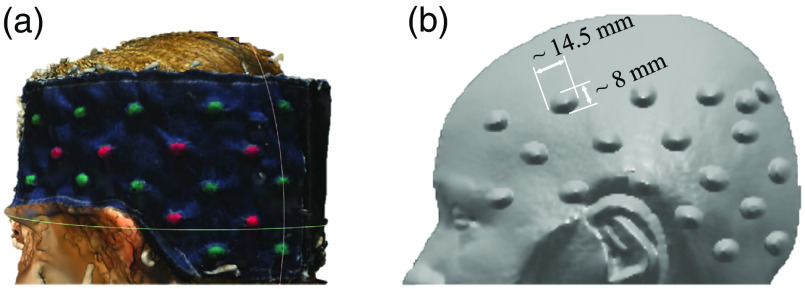
Reconstructed head images with optode-set by (a) POR and (b) MRI registration methods.

**Fig. 4:**
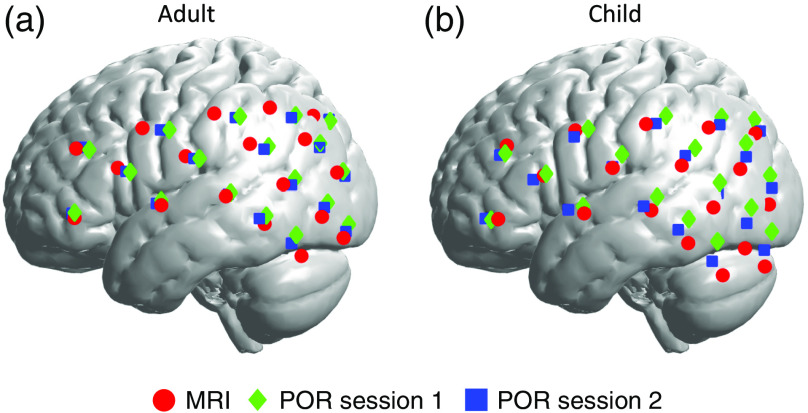
Registration results of pre-MRI, post-MRI, MRI registration sessions for both (a) adult and (b) child groups.

**Fig. 5:**
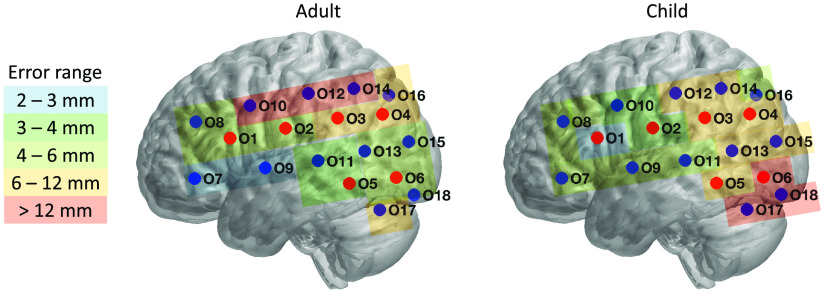
Optode-specific distance between the positions estimated by the two registration methods.

First, we estimated the MNI location of each optode for each person and registration method. Fig. 3 exemplifies a representative participant’s head image with vitamin-E marked optodes as reconstructed by both POR and MRI registration methods. Data drawn from those images served as the basis for between-method registration comparison, as detailed in the methods section. Figure 4 and Table 1 show the average MNI locations of POR and MRI registration methods for adults and children. Figure 5 and Table 1 indicate the individualized between-method deviation for optodes. We found that, in general, the optodes in the front of the cap (positive y direction) had a smaller between-method deviation, whereas the optodes in the back of the cap had a larger between-method deviation (negative y, positive/negative z direction). The optodes with the lowest deviation were optodes 7 and 9 (2 and 2.4 mm) for the adult group and optode 1 (2 mm) for the child group. The optodes with the highest deviation were optodes 10, 12, and 14 (11.4, 12.2, and 12.4 mm) for adults, and optodes 6, 17, and 18 for children (16.4, 13.6, and 12.3 mm).

**Table 1:** Averaged coordinates for all optodes within each participant group in MNI space.

Adult										
	POR (pre- and post-averaged)			MRI			Difference			
Optode	X	Y	Z	X	Y	Z	X	Y	Z	d
1	−61	34	−7	−59	39	−5	2	5	2	5.7
2	−67	1	−1	−66	6	1	1	5	2	5.5
3	−62	−32	5	−65	−25	7	3	7	2	7.9
4	−46	−58	6	−51	−51	9	5	7	3	9.1
5	−65	−31	−29	−64	−31	−32	1	0	3	3.2
6	−48	−61	−23	−49	−59	−28	1	2	5	5.5
7	−53	60	−27	−53	60	−29	0	0	2	2.0
8	−48	54	4	−47	59	4	1	5	0	5.1
9	−63	19	−21	−64	18	−23	1	1	2	2.4
10	−61	16	13	−58	27	14	3	11	1	11.4
11	−68	−15	−17	−69	−13	−19	1	2	2	3.0
12	−62	−19	19	−62	−7	21	0	12	2	12.2
13	−58	−45	−12	−61	−40	−13	3	5	1	5.9
14	−51	−45	20	−55	−34	24	4	11	4	12.4
15	−37	−70	−8	−41	−66	−7	4	4	1	5.7
16	−33	−63	18	−38	−55	20	5	8	2	9.6
17	−56	−46	−39	−52	−49	−47	4	3	8	9.4
18	−37	−71	−34	−38	−69	−39	1	2	5	5.5
Child										
1	−59	41	−9	−59	39	−9	0	2	0	2.0
2	−66	4	−2	−66	6	−5	0	2	3	3.6
3	−63	−31	3	−65	−27	−4	2	4	7	8.3
4	−47	−58	4	−50	−55	−6	3	3	10	10.9
5	−65	−28	−32	−62	−30	−41	3	2	9	9.7
6	−49	−60	−28	−46	−58	−44	3	2	16	16.4
7	−50	66	−30	−52	61	−30	2	5	0	5.4
8	−48	59	2	−47	57	6	1	2	4	4.6
9	−62	24	−24	−63	19	−27	1	5	3	5.9
10	−60	21	12	−59	24	13	1	3	1	3.3
11	−68	−12	−21	−68	−12	−25	0	0	4	4.0
12	−63	−17	18	−64	−10	16	1	7	2	7.3
13	−56	−45	−15	−59	−42	−24	3	3	9	9.9
14	−52	−46	18	−55	−40	14	3	6	4	7.8
15	−38	−70	−12	−39	−69	−22	1	1	10	10.1
16	−34	−64	15	−36	−63	12	2	1	3	3.7
17	−56	−43	−45	−51	−47	−57	5	4	12	13.6
18	−37	−69	−40	−35	−67	−52	2	2	12	12.3

**Fig. 6:**
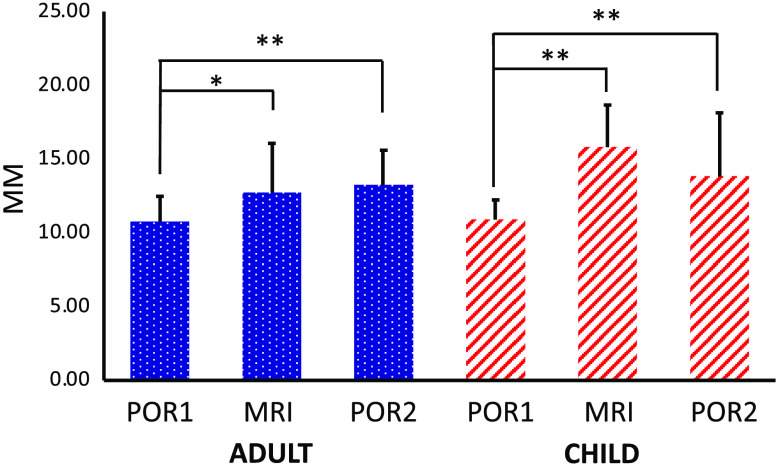
Calculated Csd of pre-MRI, post-MRI, and MRI registration sessions for both adult and child groups.

As a second step, we estimated the Csd of the three registration sessions between participants within each group. As can be seen in Fig. 6, the pre-MRI POR session gained the smallest Csd for both groups, followed by MRI and then post-MRI POR registration for adults and by post-MRI and then MRI registrations for children (pre-MRI POR adults M=10.76, SD=1.77; children M=10.91, SD=1.37; MRI adults M=12.74, SD=3.43; children M=15.81, SD=2.94; post-MRI POR adults M=13.23, SD=2.44; and children M=13.82, SD=4.44). We further conducted two ANOVA analyses on the Csd values of the three registration sessions and found significant differences within both groups [adults: F(df)=4.43 (2, 51), p=0.017; children: F(df)=10.88 (2, 51), p=0.0001]. *Post hoc* comparisons thresholded at p<0.05, Bonferroni corrected, confirmed the least amount of variability in the pre-MRI POR registration session. In particular, in both groups there was greater variability for MRI than pre-MRI POR session (adults: t=2.57, p=0.02 and children: t=7.31, p=0.000001), as well as greater variability in post-MRI POR than pre-MRI POR registration session (adults: t=4.10, p=0.0007 and children: t=2.91, p=0.01).

Third, we calculated the within-person displacement, or the difference in MNI location, between the MRI and two POR registrations methods. On average, across the axes (the x−y−z axes), the displacements were 6.66 mm for adults and 7.75 mm for children. The axis-specific displacements between MRI and POR registration sessions are as follows. MRI and pre-MRI POR for adults/children: x=2.35/1.95  mm, y=5.57/3.29  mm, and z=2.94/8.3  mm; MRI and post-MRI POR for adults/children: x=1.93/1.98  mm, y=4.59/3.23  mm, and z=2.33/4.21  mm; MRI and averaged POR for adults/children: x=2.07/1.92  mm, y=5.03/2.92  mm, and z=2.55/6.13  mm.

**Fig. 7:**
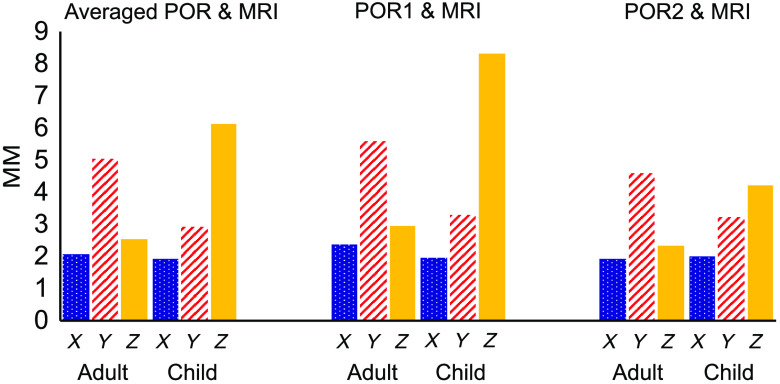
The averaged displacement of all optodes along three axes (x, y, and z axes in MNI space) between MRI and pre/post-MRI POR registrations.

The fact that the displacement between MRI and individual POR sessions was greater in the Y and Z axes than the X axis, combined with our informal session observations, suggests that the participants’ MRI cap shifted upward and forward as the participants made themselves comfortable in the supine MRI environment. Upon exiting the scanner, the shift corrected itself, but not entirely. As a result, and as can be seen in Fig. 7, POR 2 was a better match to MRI than POR 1, and the average of the two POR sessions was best at capturing the localization, likely due to the MRI-based probe shift.

NIRS data analyses rely on data channels that are centered at midpoints between the emitter–detector optode pairs. Therefore, the fourth set of analyses focused on the data channel measurement overlap revealed by the POR and MRI registrations. The average overlap was 59% for adults and 46% for children across all channels. We averaged POR sessions across the pre- and post-MRI measurements due to the potential shifts in the cap during the MRI session, as described in the section above.

**Fig. 8:**
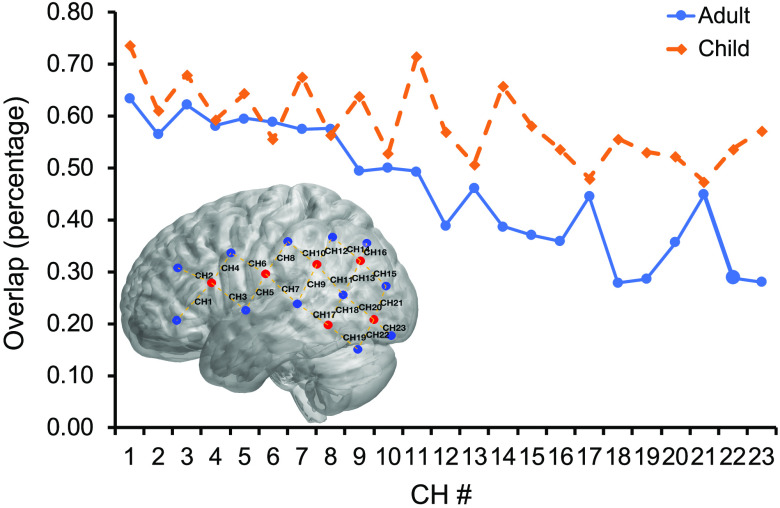
Estimated overlap of all data channels by MRI scanning and POR methods for both child and adult groups.

As can be seen in Fig. 8, there is a general trend in the reduction of overlap for both groups between the MRI and POR methods as the channel number gets bigger—which corresponds to the change in location from frontal to more posterior regions of coverage. This trend is logical considering that being supine in the MRI scanner potentially resulted in cap shifting, especially for the posterior regions closer to the back of the head where supportive cushions are placed in the MRI head coil. Therefore, considering the frontal channels (channels 1 to 4) least affected by the MRI shift and best reflective of the intended optode positioning, the average overlap was 65% for adults and 60% for children.

In the fifth and final step, we further estimated the brain regions that best corresponded with these four frontal channels (channels 1 to 4) using Monte-Carlo simulation. Table 2 presents registration results for the four frontal regions of interest. Note that, by and large, the two methods yield similar anatomical loci. There is, however, a difference in channel 4 in which only the POR method yielded precentral gyrus and in channel 1 in which only the POR method yielded Middle frontal gyrus for children.

**Table 2:** The registration results of channels 1 to 4 by both MRI scanning and POR methods, including coordinate values in MNI space and detected region estimation.

COI	MRI				POR						
	MNI coordinates			Region	MNI coordinates			Region	Difference		
	X	Y	Z		X	Y	Z		X	Y	Z
Adult											
CH1	−56	50	−17	Inferior frontal gyrus	−57	47	−17	Inferior frontal gyrus	1	3	0
CH2	−53	49	−1	Middle frontal gyrus	−55	44	−2	Inferior frontal gyrus	2	5	1
				Inferior frontal gyrus				Middle frontal gyrus			
CH3	−62	29	−14	Precentral gyrus	−62	27	−14	Precentral gyrus	0	2	0
				Superior temporal gyrus				Superior temporal gyrus			
				Inferior frontal gyrus				Inferior frontal gyrus			
CH4	−59	33	5	Inferior frontal gyrus	−61	25	3	Inferior frontal gyrus	2	8	2
								Precentral gyrus			
Child											
CH1	−56	50	−20	Inferior frontal gyrus	−55	54	−20	Inferior frontal gyrus	1	4	0
								Middle frontal gyrus			
CH2	−53	48	−2	Middle frontal gyrus	−54	50	−4	Inferior frontal gyrus	1	2	2
				Inferior frontal gyrus				Middle frontal gyrus			
CH3	−61	29	−18	Precentral gyrus	−61	33	−17	Precentral gyrus	0	4	1
				Superior temporal gyrus				Superior temporal gyrus			
				Inferior frontal gyrus				Inferior frontal gyrus			
CH4	−59	32	2	Inferior frontal gyrus	−60	31	2	Inferior frontal gyrus	1	1	0
				Precentral gyrus				Precentral gyrus			

## 4 Discussion

fNIRS is a neuroimaging method that is becoming an increasingly popular tool in the neuroimaging research community. Yet, one of its largest drawbacks is the lack of accuracy in the neuroanatomical localization. To address this major concern, we developed and explored a photogrammetry-based fNIRS optode registration method by comparing it with the gold-standard MRI registration method. The comparison revealed several key findings. We found that the Csds of pre-MRI POR registration session were smallest among all three registration sessions for both adults (10.76 mm) and children (10.91 mm). These comparison results suggest that the between participant variation during the first POR session was relatively smaller and that the increase in this variation across the subsequently administered MRI and post-MRI POR registrations was likely due to the process of getting in and out of the MRI scanner. Second, we observed that the difference between POR and MRI-based registrations was the smallest along the x axis for both adults (2.35 mm) and children (1.95 mm). In addition, we calculated the between-method optode-specific deviation. We found that the optodes in the front achieved less deviation compared with the optodes in the back, ranging from 2 mm to more than 12 mm. Finally, we observed that the optical measurement range for fNIRS data channels between POR and MRI-based registrations yielded a substantial intersection in both adults (58%) and children (46%). In addition, we further confirmed that such an intersection led to a similar estimation of cortical areas under the four frontal channels of interest across the two methods. Taken together, the findings suggest that the proposed POR method can support anatomical registration for fNIRS and other surface-based neuroimaging methods. The previous EEG electrode registration study reported a registration error of <1  mm.^18^ In our study, we were not able to achieve the same level of 1 mm registration error as this was done in an ecologically valid environment with adults and children. Nevertheless, we consider this POR approach a capable high-quality registration method for the reasons discussed below.

Our first observation revealed that the pre-MRI POR registration gained a Csd value of 10.76 (±1.77)  mm for adults and 10.90 (±1.37)  mm for children in Euclidean space. The Csd value reflects the between-participant variability or measurement error within the method. In this study, the Csd in the pre-MRI POR session, or the first registration session in our protocol, had significantly less variation in both participant groups, compared with both MRI (adult: 12.74±3.43 and child: 15.81±2.94) and post-MRI POR (adult: 13.23±2.44 and child: 13.82±4.44) sessions that followed the first POR session. The increase suggests possible cap deformation when participants were getting in and out of the MRI scanner. However, the largest Csd of 15.81 (±2.94)  mm during the MRI registration session for children was at a comparable level of 18 mm, as reported in the previous study.^29^ The observed level of variability for the pre-MRI POR registration is larger than the 4.7 to 7 mm variability reported in previous research using 3-D magnetic digitization,^25^ suggesting that there is room for improvement even for the best measurement in this study. Nevertheless, all our registration methods achieved similar registration accuracy to previous MRI work that used a registered anatomical brain atlas (18 mm variation) reported by Cooper et al.^29^ In summary, the cap movement associated with getting in and out of the head-coil might be a significant source of noise when trying to register with MRI, suggesting that 3-D magnetic digitization and photogrammetry may offer more meaningful contributions to surface-based neuroimaging method registration.

fNIRS measurements are typically taken at 25 to 30 mm distances between the optode and the detector. In this study, we achieved an average between-method error of 6.66 mm for adults and 7.75 mm for children across all optodes. To further analyze these deviations, we conducted an optode-specific between-method error calculation. As indicated in Table 1 and Fig. 5, we observed variations in these errors. For example, optodes 7 and 9 in the adult group had errors of 2.0 and 2.4 mm, respectively, and in the child group, optode 1 had an error of 2.0 mm. These low errors in both groups suggest that our method can potentially achieve a low registration error of around 2 mm. In this study, however, even though our between-method comparison suggested a relatively small displacement in the x axis of 2.35 mm for adults and 1.95 mm for children, as indicated in Fig. 7, there was a large displacement in the y axis of 5 mm for adults and in the z axis of 7 mm for children. Such differences suggested a potential cap distortion along the y and z axes in both groups when the participants entered the MRI scanner. Compared with the y and z axes, the results for the x axis are notable because those measurements were least affected by cap squeezing against head-coil cushions during MRI imaging. Nevertheless, such distortion affected a limited number of optodes, primarily in the back of the cap (e.g., optodes 10, 12, and 14 for adult and optodes 6, 17, and 18 for child), and led to errors higher than 6 mm. This approach of prioritizing the x axis is further motivated by an MRI-based registration study that compared the performance of the MNI and Talairach registration methods and discovered less displacement along the x and y axes than the z axis.^13^ The better performance of the *y* axis in this previous work was likely because they only had optodes on the frontal lobe. In summary, to the best of our knowledge, this is the only paper to report a direct contrast between two surface-based registration methods along multiple dimensions, and it suggests both an acceptable as well as a superior performance of the POR method relative to the MRI registration.

In the fNIRS approach, a measurement channel is defined as the mid-point between the light emitter and detector. In this study, we employed a set of overlap calculations to examine the consistency between the two registration methods for each data channel. The average across all channels was 59% for adults and 46% for children. The overlap was stronger for the frontal regions that were least affected by the MRI-based procedure, yielding 65% for adults and 60% for children. These overlap values can be interpreted as, in average, $19.6\;{\mathrm{cm}}^{3}$ for adult and $15.4\;{\mathrm{cm}}^{3}$ for child out of $33.5\;{\mathrm{cm}}^{3}$ were mutual measurement areas localized by the two registration methods, suggesting a substantial overlap between the two methods’ registration results for the same fNIRS optode set. This conclusion was further confirmed by our estimations suggesting that the frontal channels also maximally covered similar brain regions of interest (Table 2). In our work, we used Monte-Carlo simulation based on a simple spherical model for describing and examining the measurement overlap for fNIRS data channels. An alternative and more thorough way of examining probability of photon distribution, the Monte Carlo photon migration simulation method, can be found in previous localization works for fNIRS.^10^^,^^29^

Finally, hair is a common concern in fNIRS research. With regard to localization estimates, hair thickness and volume may cause distortions in cap sizes. The present method addresses this issue by combing and adjusting the participants hair, so it does not interfere with the optodes before cap wearing. To avoid volume distortions, research must also consider the choice of material in cap design.

To conclude, we developed the POR method to overcome several obstacles associated with current registration methods. First, unlike MRI and 3-D digitization methods, the POR is not affected by metal or magnetic field surrounding the measurements. Second, it is more accessible and cost effective than either 3-D or MRI measurements. Finally, unlike the MRI, the POR does not touch or otherwise affect the positioning of the headgear. The findings suggest that the POR method can yield easily accessible and meaningful anatomical registration for fNIRS and possibly other surface-based neuroimaging methods.
